# 1,3-Diphenyl-1*H*-pyrazole-4-carbaldehyde

**DOI:** 10.1107/S1600536810045630

**Published:** 2010-11-13

**Authors:** Abdul Qayyum Ather, M. Nawaz Tahir, Misbahul Ain Khan, Karamat Mehmood, Faryal Chaudhry

**Affiliations:** a Department of Chemistry, Islamia University, Bahawalpur, Pakistan; b Applied Chemistry Research Center, PCSIR Laboratories Complex, Lahore 54600, Pakistan; c Department of Physics, University of Sargodha, Sargodha, Pakistan; d Institute of Chemistry, University of the Punjab, Lahore, Pakistan

## Abstract

There are four mol­ecules in the asymmetric unit of the title compound, C_16_H_12_N_2_O. The dihedral angle between the phenyl rings in the mol­ecules are 22.2 (2), 22.4 (2), 25.1 (3) and 41.9 (2)°. In the crystal, mol­ecules form dimers due to inter­molecular C—H⋯O hydrogen bonds, which result in one *R*_2_^2^(10) and two *R*_2_^1^(7) ring motifs. Weak aromatic π–π stacking [centroid–centroid separation = 3.788 (3) Å] and C—H⋯π inter­actions may also consolidate the packing.

## Related literature

For background and related structures, see: Ather *et al.* (2010**a* ▶,*b* ▶,*c* ▶,d* ▶). For graph-set notation, see: Bernstein *et al.* (1995 ▶).
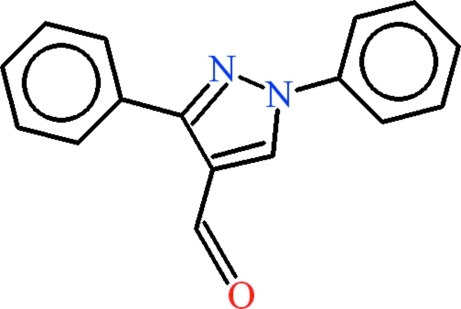


## Experimental

### 

#### Crystal data

C_16_H_12_N_2_O
*M* *_r_* = 248.28
Triclinic, 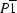

*a* = 10.1367 (9) Å
*b* = 15.5952 (16) Å
*c* = 16.7550 (15) Å
α = 95.932 (6)°
β = 90.135 (5)°
γ = 107.991 (6)°
*V* = 2504.1 (4) Å^3^
*Z* = 8
Mo *K*α radiation
μ = 0.08 mm^−1^
*T* = 296 K
0.32 × 0.16 × 0.14 mm

#### Data collection

Bruker Kappa APEXII CCD diffractometer
Absorption correction: multi-scan (*SADABS*; Bruker, 2005 ▶) *T*_min_ = 0.982, *T*_max_ = 0.988
34716 measured reflections
8912 independent reflections
4643 reflections with *I* > 2σ(*I*)
*R*_int_ = 0.092

#### Refinement

*R*[*F* ^2^ > 2σ(*F* ^2^)] = 0.090
*wR*(*F* ^2^) = 0.259
*S* = 1.04
8912 reflections
671 parameters
H-atom parameters constrained
Δρ_max_ = 0.26 e Å^−3^
Δρ_min_ = −0.26 e Å^−3^

### 

Data collection: *APEX2* (Bruker, 2009 ▶); cell refinement: *SAINT* (Bruker, 2009 ▶); data reduction: *SAINT*; program(s) used to solve structure: *SHELXS97* (Sheldrick, 2008 ▶); program(s) used to refine structure: *SHELXL97* (Sheldrick, 2008 ▶); molecular graphics: *ORTEP-3 for Windows* (Farrugia, 1997 ▶) and *PLATON* (Spek, 2009 ▶); software used to prepare material for publication: *WinGX* (Farrugia, 1999 ▶) and *PLATON*.

## Supplementary Material

Crystal structure: contains datablocks global, I. DOI: 10.1107/S1600536810045630/hb5729sup1.cif

Structure factors: contains datablocks I. DOI: 10.1107/S1600536810045630/hb5729Isup2.hkl

Additional supplementary materials:  crystallographic information; 3D view; checkCIF report

## Figures and Tables

**Table 1:** Hydrogen-bond geometry (Å, °) *Cg*5 is the centroid of the C17–C22 ring.

*D*—H⋯*A*	*D*—H	H⋯*A*	*D*⋯*A*	*D*—H⋯*A*
C10—H10⋯O2^i^	0.93	2.38	3.299 (5)	168
C12—H12⋯O2^i^	0.93	2.45	3.373 (6)	175
C26—H26⋯O1^i^	0.93	2.38	3.298 (6)	169
C32—H32⋯O1^i^	0.93	2.55	3.423 (7)	156
C42—H42⋯O3^ii^	0.93	2.36	3.287 (7)	173
C44—H44⋯O3^ii^	0.93	2.57	3.495 (8)	175
C58—H58⋯O4^iii^	0.93	2.44	3.353 (6)	165
C60—H60⋯O4^iii^	0.93	2.49	3.330 (8)	150
C2—H2⋯*Cg*5^iv^	0.93	2.86	3.689 (7)	149

Symmetry codes: (i) 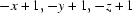; (ii) 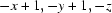; (iii) 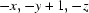; (iv) 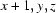.

**Fig. 1.**
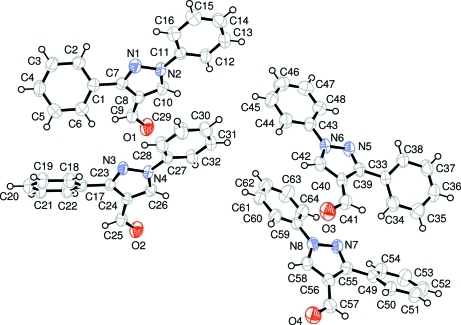
View of the title compound with displacement ellipsoids drawn at the 50% probability level.

**Fig. 2.**
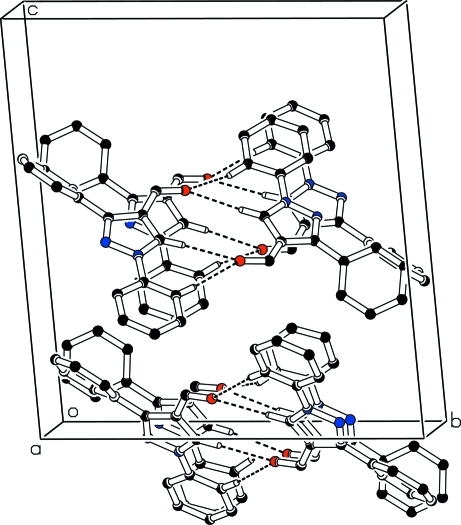
Packing diagram of the title compound, showing that molecules are dimerized.

## supplementary crystallographic information

### Comment

In continuation of our studies of pyrazole derivatives (Ather *et al.*, 2010**a*,*b*,*c*,d*), the title compound (I, Fig. 1) is being reported here.

The title compound consists of four molecules in the crystallographic asymmetric unit which differ from each other geometrically. In one molecule, the phenyl rings A (C1—C6), B (C11—C16) and pyrazole moiety C (C7—C11/N1/N2/O1) are planar with r. m. s. deviation of 0.0048, 0.0057 and 0.0178 Å, respectively. The dihedral angle between A/B, A/C and B/C is 41.93 (22), 43.62 (21)° and 1.97 (31)°, respectively. In second molecule, the phenyl rings D (C17—C22), E (C27—C32) and pyrazole moiety F (C23—C26/N3/N4/O2) are planar with r. m. s. deviation of 0.0046, 0.0067 and 0.0330 Å, respectively. The dihedral angle between D/E, D/F and E/F is 22.38 (22), 34.73 (15)° and 17.24 (25)°, respectively. These two molecules form dimers due to intermolecular H-bondings of C—H···O type with two *R*_2_^1^(7) and an *R*_2_^2^(10) (Table 1, Fig. 2) ring motifs (Bernstein *et al.*, 1995). In third molecule, the phenyl rings G (C33—C38), H (C43—C48) and pyrazole moiety I (C39—C42/N5/N6/O3) are planar with r. m. s. deviation of 0.0099, 0.0021 and 0.0292 Å, respectively. The dihedral angle between G/H, G/I and H/I is 25.07 (28), 27.78 (27)° and 3.81 (33)°, respectively. In fourth molecule, the phenyl rings J (C49—C54), K (C59—C64) and pyrazole moiety *L* (C55—C58/N7/N8/O4) are planar with r. m. s. deviation of 0.0049, 0.0029 and 0.0339 Å, respectively. The dihedral angle between J/K, J/*L* and K/*L* is 22.16 (24), 37.81 (18)° and 22.27 (25)°, respectively. Third and fourth molecules also form dimers due to intermolecular H-bondings of C—H···O type with two *R*_2_^1^(7) and an *R*_2_^2^(10) (Table 1, Fig. 2). π···π interactions occurs between the pyrazole and benzene rings in each dimer. The separations between the centroids of pyrazole and benzene rings have values of 3.788 (3)Å. These π···π and C—H···π (Table 1) interactions may help to consolidate the packing.

### Experimental

Phosphoryl chloride (5 ml) was added drop wise to cold *N*,*N*-dimethylformamide (DMF) (15 ml) with continuous stirring at 273–278 K for about 30 min. Acetophenon phenylhydrazone (3.15 g, 15 mmol) was separately dissolved in 5 ml of DMF and was added drop wise to the former cold mixture with the continuous stirring at 273–278 K for an hour. The resulting mixture was further stirred at 323–333 K for 5–6 h and cooled to room temperature. The crude product was poured into crushed ice which resulted into white precipitate. These precipitate were recrystallized in ethanol to obtain colorless needles of (I).

### Refinement

The H-atoms were positioned geometrically (C–H = 0.93 Å) and refined as riding with *U*_iso_(H) = *xU*_eq_(C), where *x* = 1.2 for all H-atoms.

### Crystal data

**Table:**

C_16_H_12_N_2_O	*Z* = 8
*M**_r_* = 248.28	*F*(000) = 1040
Triclinic, *P*1	*D*_x_ = 1.317 Mg m^−^^3^
Hall symbol: -P 1	Mo *K*α radiation, λ = 0.71073 Å
*a* = 10.1367 (9) Å	Cell parameters from 4643 reflections
*b* = 15.5952 (16) Å	θ = 1.2–25.1°
*c* = 16.7550 (15) Å	µ = 0.08 mm^−^^1^
α = 95.932 (6)°	*T* = 296 K
β = 90.135 (5)°	Needle, colorless
γ = 107.991 (6)°	0.32 × 0.16 × 0.14 mm
*V* = 2504.1 (4) Å^3^	

### Data collection

**Table:**

Bruker Kappa APEXII CCD diffractometer	8912 independent reflections
Radiation source: fine-focus sealed tube	4643 reflections with *I* > 2σ(*I*)
graphite	*R*_int_ = 0.092
Detector resolution: 8.3 pixels mm^-1^	θ_max_ = 25.1°, θ_min_ = 1.2°
ω scans	*h* = −12→11
Absorption correction: multi-scan (*SADABS*; Bruker, 2005)	*k* = −18→18
*T*_min_ = 0.982, *T*_max_ = 0.988	*l* = −20→20
34716 measured reflections	

### Refinement

**Table:**

Refinement on *F*^2^	Secondary atom site location: difference Fourier map
Least-squares matrix: full	Hydrogen site location: inferred from neighbouring sites
*R*[*F*^2^ > 2σ(*F*^2^)] = 0.090	H-atom parameters constrained
*wR*(*F*^2^) = 0.259	*w* = 1/[σ^2^(*F*_o_^2^) + (0.0599*P*)^2^ + 5.7411*P*] where *P* = (*F*_o_^2^ + 2*F*_c_^2^)/3
*S* = 1.04	(Δ/σ)_max_ < 0.001
8912 reflections	Δρ_max_ = 0.26 e Å^−^^3^
671 parameters	Δρ_min_ = −0.25 e Å^−^^3^
0 restraints	Extinction correction: *SHELXL97* (Sheldrick, 2008), Fc^*^=kFc[1+0.001xFc^2^λ^3^/sin(2θ)]^-1/4^
Primary atom site location: structure-invariant direct methods	Extinction coefficient: 0.0043 (7)

### Special details

**Table:**

Geometry. Bond distances, angles *etc*. have been calculated using the rounded fractional coordinates. All su's are estimated from the variances of the (full) variance-covariance matrix. The cell e.s.d.'s are taken into account in the estimation of distances, angles and torsion angles
Refinement. Refinement of *F*^2^ against ALL reflections. The weighted *R*-factor *wR* and goodness of fit *S* are based on *F*^2^, conventional *R*-factors *R* are based on *F*, with *F* set to zero for negative *F*^2^. The threshold expression of *F*^2^ > σ(*F*^2^) is used only for calculating *R*-factors(gt) *etc*. and is not relevant to the choice of reflections for refinement. *R*-factors based on *F*^2^ are statistically about twice as large as those based on *F*, and *R*- factors based on ALL data will be even larger.

### Fractional atomic coordinates and isotropic or equivalent isotropic displacement parameters (Å^2^)

**Table:**

	*x*	*y*	*z*	*U*_iso_*/*U*_eq_	
O1	0.6324 (5)	0.4151 (3)	0.5760 (3)	0.0706 (16)	
N1	0.8394 (4)	0.2089 (3)	0.4618 (3)	0.0490 (17)	
N2	0.8812 (4)	0.2918 (3)	0.4319 (2)	0.0428 (14)	
C1	0.6788 (5)	0.1383 (3)	0.5612 (3)	0.0446 (17)	
C2	0.7562 (6)	0.0892 (3)	0.5903 (3)	0.0493 (17)	
C3	0.6934 (6)	0.0185 (4)	0.6334 (3)	0.061 (2)	
C4	0.5531 (7)	−0.0053 (4)	0.6477 (4)	0.065 (2)	
C5	0.4758 (7)	0.0432 (4)	0.6173 (4)	0.076 (3)	
C6	0.5391 (6)	0.1157 (4)	0.5744 (4)	0.062 (2)	
C7	0.7457 (5)	0.2158 (3)	0.5157 (3)	0.0436 (17)	
C8	0.7289 (5)	0.3034 (3)	0.5215 (3)	0.0429 (17)	
C9	0.6454 (6)	0.3399 (4)	0.5749 (3)	0.053 (2)	
C10	0.8163 (5)	0.3480 (3)	0.4661 (3)	0.0470 (17)	
C11	0.9833 (5)	0.3067 (3)	0.3719 (3)	0.0437 (17)	
C12	1.0281 (6)	0.3897 (4)	0.3421 (3)	0.055 (2)	
C13	1.1282 (7)	0.4025 (4)	0.2846 (4)	0.068 (2)	
C14	1.1816 (6)	0.3351 (4)	0.2571 (3)	0.061 (2)	
C15	1.1377 (6)	0.2537 (4)	0.2883 (3)	0.061 (2)	
C16	1.03802 (19)	0.23882 (12)	0.34566 (11)	0.0506 (17)	
O2	0.09209 (19)	0.44158 (12)	0.58037 (11)	0.0644 (16)	
N3	0.33043 (19)	0.24537 (12)	0.48630 (11)	0.0433 (16)	
N4	0.36575 (19)	0.32530 (12)	0.45286 (11)	0.0405 (14)	
C17	0.17474 (19)	0.17575 (12)	0.58564 (11)	0.0384 (17)	
C18	0.15852 (19)	0.08767 (12)	0.55248 (11)	0.0502 (19)	
C19	0.1044 (6)	0.0147 (4)	0.5952 (3)	0.055 (2)	
C20	0.0655 (5)	0.0274 (3)	0.6720 (3)	0.0508 (19)	
C21	0.0812 (6)	0.1139 (4)	0.7069 (3)	0.0531 (19)	
C22	0.1365 (5)	0.1882 (3)	0.6647 (3)	0.0484 (17)	
C23	0.2357 (5)	0.2514 (3)	0.5386 (3)	0.0396 (17)	
C24	0.2102 (5)	0.3372 (3)	0.5403 (3)	0.0393 (17)	
C25	0.1049 (5)	0.3669 (4)	0.5811 (3)	0.0469 (17)	
C26	0.2954 (5)	0.3806 (3)	0.4830 (3)	0.0438 (17)	
C27	0.4626 (5)	0.3384 (3)	0.3899 (3)	0.0402 (17)	
C28	0.4935 (6)	0.2652 (4)	0.3532 (4)	0.061 (2)	
C29	0.5852 (7)	0.2762 (4)	0.2916 (4)	0.069 (3)	
C30	0.6470 (6)	0.3599 (4)	0.2681 (3)	0.060 (2)	
C31	0.6156 (6)	0.4331 (4)	0.3051 (3)	0.056 (2)	
C32	0.5242 (5)	0.4236 (4)	0.3679 (3)	0.0517 (19)	
O3	0.4419 (4)	0.5710 (3)	−0.0773 (3)	0.0691 (16)	
N5	0.8514 (4)	0.7740 (3)	0.0322 (3)	0.0502 (17)	
N6	0.8115 (4)	0.6927 (3)	0.0632 (2)	0.0444 (14)	
C33	0.7671 (5)	0.8451 (3)	−0.0696 (3)	0.0460 (17)	
C34	0.6511 (6)	0.8609 (4)	−0.1006 (3)	0.061 (2)	
C35	0.6656 (7)	0.9337 (4)	−0.1449 (4)	0.064 (2)	
C36	0.7956 (7)	0.9910 (4)	−0.1562 (4)	0.066 (3)	
C37	0.9105 (7)	0.9786 (4)	−0.1242 (4)	0.069 (3)	
C38	0.8963 (6)	0.9042 (4)	−0.0818 (3)	0.057 (2)	
C39	0.7537 (5)	0.7677 (3)	−0.0235 (3)	0.0455 (17)	
C40	0.6495 (5)	0.6820 (3)	−0.0279 (3)	0.0435 (17)	
C41	0.5301 (6)	0.6432 (4)	−0.0824 (3)	0.055 (2)	
C42	0.6925 (5)	0.6367 (4)	0.0296 (3)	0.0494 (17)	
C43	0.9001 (5)	0.6761 (4)	0.1228 (3)	0.0466 (17)	
C44	0.8628 (6)	0.5944 (4)	0.1538 (3)	0.059 (2)	
C45	0.9511 (7)	0.5794 (5)	0.2108 (4)	0.069 (3)	
C46	1.0713 (7)	0.6456 (5)	0.2354 (3)	0.066 (3)	
C47	1.1076 (6)	0.7277 (4)	0.2040 (4)	0.063 (2)	
C48	1.0217 (6)	0.7430 (4)	0.1472 (3)	0.054 (2)	
O4	−0.0749 (4)	0.5732 (3)	−0.0812 (3)	0.0707 (17)	
N7	0.3455 (4)	0.7707 (3)	0.0177 (3)	0.0483 (17)	
N8	0.3045 (4)	0.6894 (3)	0.0502 (3)	0.0463 (17)	
C49	0.2593 (5)	0.8414 (3)	−0.0837 (3)	0.0439 (17)	
C50	0.2157 (5)	0.8280 (4)	−0.1632 (3)	0.0493 (17)	
C51	0.2342 (6)	0.9014 (4)	−0.2059 (3)	0.060 (2)	
C52	0.2942 (6)	0.9890 (4)	−0.1697 (4)	0.065 (2)	
C53	0.3354 (7)	1.0013 (4)	−0.0901 (4)	0.067 (2)	
C54	0.3184 (6)	0.9292 (4)	−0.0465 (3)	0.0577 (19)	
C55	0.2457 (5)	0.7637 (3)	−0.0361 (3)	0.0435 (17)	
C56	0.1416 (5)	0.6776 (3)	−0.0397 (3)	0.0440 (17)	
C57	0.0096 (6)	0.6466 (4)	−0.0828 (3)	0.0523 (19)	
C58	0.1845 (5)	0.6332 (3)	0.0175 (3)	0.0442 (17)	
C59	0.3854 (5)	0.6767 (4)	0.1152 (3)	0.0456 (17)	
C60	0.3706 (6)	0.5903 (4)	0.1348 (3)	0.0546 (19)	
C61	0.4465 (6)	0.5811 (4)	0.1997 (3)	0.060 (2)	
C62	0.5361 (6)	0.6533 (4)	0.2435 (4)	0.065 (2)	
C63	0.5492 (7)	0.7385 (4)	0.2227 (4)	0.075 (3)	
C64	0.4736 (6)	0.7498 (4)	0.1583 (4)	0.066 (2)	
H2	0.85051	0.10389	0.58087	0.0589*	
H3	0.74626	−0.01426	0.65353	0.0725*	
H4	0.51177	−0.05327	0.67733	0.0771*	
H5	0.38094	0.02736	0.62541	0.0908*	
H6	0.48683	0.14908	0.55469	0.0745*	
H9	0.59662	0.30309	0.61223	0.0640*	
H10	0.82820	0.40689	0.45441	0.0562*	
H12	0.99197	0.43596	0.36019	0.0659*	
H13	1.15977	0.45823	0.26427	0.0811*	
H14	1.24721	0.34440	0.21758	0.0732*	
H15	1.17538	0.20802	0.27071	0.0726*	
H16	1.00809	0.18326	0.36646	0.0602*	
H18	0.18484	0.07771	0.50008	0.0602*	
H19	0.09443	−0.04369	0.57147	0.0659*	
H20	0.02851	−0.02200	0.70070	0.0608*	
H21	0.05453	0.12268	0.75942	0.0637*	
H22	0.14805	0.24639	0.68924	0.0584*	
H25	0.04212	0.32593	0.61009	0.0562*	
H26	0.30279	0.43768	0.46808	0.0525*	
H28	0.45295	0.20797	0.36967	0.0730*	
H29	0.60499	0.22610	0.26589	0.0828*	
H30	0.71008	0.36723	0.22717	0.0725*	
H31	0.65575	0.49007	0.28813	0.0670*	
H32	0.50546	0.47378	0.39433	0.0623*	
H34	0.56304	0.82262	−0.09178	0.0732*	
H35	0.58768	0.94349	−0.16675	0.0761*	
H36	0.80514	1.03927	−0.18639	0.0786*	
H37	0.99777	1.01937	−0.13051	0.0830*	
H38	0.97502	0.89429	−0.06135	0.0687*	
H41	0.52026	0.67578	−0.12427	0.0654*	
H42	0.64684	0.57838	0.04222	0.0596*	
H44	0.78001	0.54974	0.13715	0.0712*	
H45	0.92784	0.52416	0.23215	0.0822*	
H46	1.12959	0.63530	0.27372	0.0791*	
H47	1.18989	0.77259	0.22111	0.0760*	
H48	1.04565	0.79806	0.12559	0.0653*	
H50	0.17392	0.76955	−0.18798	0.0593*	
H51	0.20603	0.89206	−0.25977	0.0727*	
H52	0.30642	1.03845	−0.19869	0.0782*	
H53	0.37566	1.05987	−0.06517	0.0802*	
H54	0.34615	0.93888	0.00746	0.0697*	
H57	−0.01206	0.68650	−0.11423	0.0629*	
H58	0.13851	0.57531	0.03084	0.0530*	
H60	0.31062	0.53966	0.10490	0.0657*	
H61	0.43586	0.52325	0.21380	0.0720*	
H62	0.58753	0.64559	0.28671	0.0772*	
H63	0.60979	0.78907	0.25241	0.0903*	
H64	0.48343	0.80769	0.14466	0.0791*	

### Atomic displacement parameters (Å^2^)

**Table:**

	*U*^11^	*U*^22^	*U*^33^	*U*^12^	*U*^13^	*U*^23^
O1	0.099 (3)	0.045 (2)	0.077 (3)	0.034 (2)	0.024 (2)	0.012 (2)
N1	0.057 (3)	0.042 (3)	0.049 (3)	0.016 (2)	0.005 (2)	0.008 (2)
N2	0.054 (3)	0.036 (2)	0.039 (2)	0.013 (2)	0.006 (2)	0.0093 (19)
C1	0.049 (3)	0.043 (3)	0.040 (3)	0.012 (3)	0.000 (2)	0.004 (2)
C2	0.051 (3)	0.047 (3)	0.050 (3)	0.015 (3)	0.001 (3)	0.007 (3)
C3	0.071 (4)	0.059 (4)	0.060 (4)	0.027 (3)	0.006 (3)	0.022 (3)
C4	0.076 (4)	0.055 (4)	0.066 (4)	0.020 (3)	0.014 (3)	0.024 (3)
C5	0.053 (4)	0.078 (5)	0.101 (5)	0.019 (3)	0.021 (4)	0.035 (4)
C6	0.057 (4)	0.063 (4)	0.075 (4)	0.025 (3)	0.012 (3)	0.023 (3)
C7	0.046 (3)	0.040 (3)	0.044 (3)	0.012 (2)	−0.001 (2)	0.006 (2)
C8	0.050 (3)	0.033 (3)	0.047 (3)	0.015 (2)	0.003 (2)	0.004 (2)
C9	0.066 (4)	0.048 (4)	0.050 (3)	0.021 (3)	0.010 (3)	0.014 (3)
C10	0.060 (3)	0.040 (3)	0.043 (3)	0.017 (3)	0.002 (3)	0.009 (2)
C11	0.042 (3)	0.047 (3)	0.040 (3)	0.010 (2)	−0.003 (2)	0.008 (2)
C12	0.066 (4)	0.048 (3)	0.057 (4)	0.022 (3)	0.016 (3)	0.017 (3)
C13	0.072 (4)	0.065 (4)	0.070 (4)	0.020 (3)	0.018 (3)	0.031 (3)
C14	0.056 (4)	0.071 (4)	0.056 (4)	0.019 (3)	0.012 (3)	0.008 (3)
C15	0.065 (4)	0.057 (4)	0.056 (4)	0.017 (3)	0.005 (3)	−0.005 (3)
C16	0.057 (3)	0.041 (3)	0.050 (3)	0.012 (3)	0.001 (3)	−0.002 (3)
O2	0.086 (3)	0.046 (2)	0.070 (3)	0.032 (2)	0.015 (2)	0.010 (2)
N3	0.050 (3)	0.034 (2)	0.047 (3)	0.0119 (19)	0.005 (2)	0.013 (2)
N4	0.050 (3)	0.034 (2)	0.038 (2)	0.012 (2)	0.004 (2)	0.0101 (19)
C17	0.043 (3)	0.027 (3)	0.045 (3)	0.010 (2)	0.000 (2)	0.006 (2)
C18	0.061 (4)	0.043 (3)	0.045 (3)	0.014 (3)	0.004 (3)	0.004 (3)
C19	0.070 (4)	0.034 (3)	0.060 (4)	0.014 (3)	0.007 (3)	0.008 (3)
C20	0.058 (3)	0.036 (3)	0.058 (4)	0.010 (3)	0.006 (3)	0.018 (3)
C21	0.065 (4)	0.049 (3)	0.048 (3)	0.019 (3)	0.014 (3)	0.014 (3)
C22	0.062 (3)	0.040 (3)	0.044 (3)	0.017 (3)	0.007 (3)	0.005 (2)
C23	0.042 (3)	0.038 (3)	0.038 (3)	0.010 (2)	−0.001 (2)	0.008 (2)
C24	0.044 (3)	0.033 (3)	0.041 (3)	0.011 (2)	0.002 (2)	0.007 (2)
C25	0.057 (3)	0.043 (3)	0.041 (3)	0.016 (3)	0.005 (3)	0.005 (2)
C26	0.050 (3)	0.035 (3)	0.048 (3)	0.015 (2)	0.000 (2)	0.006 (2)
C27	0.044 (3)	0.042 (3)	0.036 (3)	0.014 (2)	0.003 (2)	0.008 (2)
C28	0.070 (4)	0.044 (3)	0.071 (4)	0.019 (3)	0.023 (3)	0.015 (3)
C29	0.087 (5)	0.058 (4)	0.067 (4)	0.028 (3)	0.031 (4)	0.009 (3)
C30	0.062 (4)	0.077 (4)	0.045 (3)	0.023 (3)	0.018 (3)	0.015 (3)
C31	0.058 (4)	0.053 (4)	0.058 (4)	0.015 (3)	0.011 (3)	0.024 (3)
C32	0.058 (3)	0.043 (3)	0.054 (4)	0.014 (3)	0.008 (3)	0.010 (3)
O3	0.072 (3)	0.046 (2)	0.075 (3)	−0.002 (2)	−0.005 (2)	0.005 (2)
N5	0.056 (3)	0.045 (3)	0.047 (3)	0.009 (2)	0.005 (2)	0.014 (2)
N6	0.050 (3)	0.040 (2)	0.038 (2)	0.005 (2)	0.007 (2)	0.008 (2)
C33	0.053 (3)	0.042 (3)	0.038 (3)	0.006 (3)	0.006 (2)	0.009 (2)
C34	0.060 (4)	0.054 (4)	0.063 (4)	0.006 (3)	0.004 (3)	0.014 (3)
C35	0.072 (4)	0.052 (4)	0.063 (4)	0.014 (3)	−0.010 (3)	0.007 (3)
C36	0.084 (5)	0.051 (4)	0.054 (4)	0.006 (3)	0.001 (3)	0.019 (3)
C37	0.064 (4)	0.058 (4)	0.076 (5)	−0.002 (3)	0.009 (3)	0.026 (3)
C38	0.054 (3)	0.056 (4)	0.058 (4)	0.008 (3)	0.002 (3)	0.019 (3)
C39	0.054 (3)	0.036 (3)	0.045 (3)	0.011 (2)	0.010 (3)	0.007 (2)
C40	0.056 (3)	0.033 (3)	0.039 (3)	0.009 (2)	0.006 (2)	0.007 (2)
C41	0.062 (4)	0.047 (3)	0.052 (4)	0.012 (3)	−0.002 (3)	0.008 (3)
C42	0.052 (3)	0.041 (3)	0.051 (3)	0.007 (3)	0.006 (3)	0.009 (3)
C43	0.055 (3)	0.049 (3)	0.036 (3)	0.017 (3)	0.005 (2)	0.003 (2)
C44	0.062 (4)	0.056 (4)	0.055 (4)	0.010 (3)	0.000 (3)	0.012 (3)
C45	0.082 (5)	0.072 (4)	0.059 (4)	0.029 (4)	0.001 (4)	0.022 (3)
C46	0.069 (4)	0.089 (5)	0.046 (4)	0.037 (4)	−0.007 (3)	0.000 (3)
C47	0.059 (4)	0.068 (4)	0.059 (4)	0.018 (3)	−0.004 (3)	−0.005 (3)
C48	0.055 (4)	0.056 (4)	0.051 (4)	0.017 (3)	0.003 (3)	0.001 (3)
O4	0.064 (3)	0.059 (3)	0.072 (3)	−0.006 (2)	−0.014 (2)	0.008 (2)
N7	0.051 (3)	0.045 (3)	0.046 (3)	0.008 (2)	0.002 (2)	0.013 (2)
N8	0.044 (3)	0.050 (3)	0.042 (3)	0.009 (2)	0.004 (2)	0.010 (2)
C49	0.044 (3)	0.044 (3)	0.043 (3)	0.013 (2)	0.002 (2)	0.004 (2)
C50	0.055 (3)	0.046 (3)	0.046 (3)	0.013 (3)	0.001 (3)	0.010 (3)
C51	0.069 (4)	0.074 (4)	0.040 (3)	0.024 (3)	−0.004 (3)	0.009 (3)
C52	0.076 (4)	0.053 (4)	0.069 (4)	0.019 (3)	0.000 (3)	0.021 (3)
C53	0.088 (5)	0.043 (3)	0.063 (4)	0.011 (3)	−0.011 (3)	0.002 (3)
C54	0.075 (4)	0.047 (3)	0.043 (3)	0.009 (3)	−0.006 (3)	0.000 (3)
C55	0.047 (3)	0.048 (3)	0.035 (3)	0.015 (3)	0.002 (2)	0.001 (2)
C56	0.045 (3)	0.040 (3)	0.045 (3)	0.011 (2)	0.005 (2)	0.003 (2)
C57	0.053 (3)	0.054 (4)	0.043 (3)	0.007 (3)	−0.001 (3)	0.004 (3)
C58	0.046 (3)	0.040 (3)	0.041 (3)	0.005 (2)	0.002 (2)	0.005 (2)
C59	0.041 (3)	0.053 (3)	0.039 (3)	0.008 (3)	0.002 (2)	0.009 (3)
C60	0.055 (3)	0.045 (3)	0.059 (4)	0.008 (3)	−0.005 (3)	0.008 (3)
C61	0.067 (4)	0.058 (4)	0.057 (4)	0.018 (3)	0.001 (3)	0.022 (3)
C62	0.065 (4)	0.065 (4)	0.060 (4)	0.012 (3)	−0.009 (3)	0.015 (3)
C63	0.091 (5)	0.051 (4)	0.071 (5)	0.006 (3)	−0.032 (4)	0.003 (3)
C64	0.072 (4)	0.048 (4)	0.067 (4)	0.002 (3)	−0.021 (3)	0.012 (3)

### Geometric parameters (Å, °)

**Table:**

O1—C9	1.218 (8)	C20—H20	0.9300
O2—C25	1.212 (6)	C21—H21	0.9300
O3—C41	1.214 (8)	C22—H22	0.9300
O4—C57	1.202 (8)	C25—H25	0.9300
N1—N2	1.378 (6)	C26—H26	0.9300
N1—C7	1.333 (7)	C28—H28	0.9300
N2—C10	1.336 (7)	C29—H29	0.9300
N2—C11	1.428 (6)	C30—H30	0.9300
N3—C23	1.320 (6)	C31—H31	0.9300
N3—N4	1.366 (3)	C32—H32	0.9300
N4—C26	1.341 (5)	C33—C39	1.471 (7)
N4—C27	1.430 (5)	C33—C38	1.379 (8)
N5—C39	1.333 (7)	C33—C34	1.384 (8)
N5—N6	1.364 (6)	C34—C35	1.390 (8)
N6—C42	1.334 (7)	C35—C36	1.371 (10)
N6—C43	1.437 (7)	C36—C37	1.356 (10)
N7—N8	1.377 (6)	C37—C38	1.393 (8)
N7—C55	1.325 (7)	C39—C40	1.419 (7)
N8—C59	1.428 (7)	C40—C41	1.447 (8)
N8—C58	1.337 (7)	C40—C42	1.395 (7)
C1—C7	1.479 (7)	C43—C48	1.375 (8)
C1—C6	1.374 (8)	C43—C44	1.370 (8)
C1—C2	1.373 (7)	C44—C45	1.393 (9)
C2—C3	1.370 (7)	C45—C46	1.361 (10)
C3—C4	1.383 (10)	C46—C47	1.379 (9)
C4—C5	1.375 (10)	C47—C48	1.376 (9)
C5—C6	1.389 (9)	C34—H34	0.9300
C7—C8	1.422 (7)	C35—H35	0.9300
C8—C10	1.374 (7)	C36—H36	0.9300
C8—C9	1.427 (8)	C37—H37	0.9300
C11—C12	1.379 (7)	C38—H38	0.9300
C11—C16	1.375 (5)	C41—H41	0.9300
C12—C13	1.385 (9)	C42—H42	0.9300
C13—C14	1.365 (9)	C44—H44	0.9300
C14—C15	1.368 (8)	C45—H45	0.9300
C15—C16	1.380 (6)	C46—H46	0.9300
C2—H2	0.9300	C47—H47	0.9300
C3—H3	0.9300	C48—H48	0.9300
C4—H4	0.9300	C49—C50	1.377 (7)
C5—H5	0.9300	C49—C54	1.392 (7)
C6—H6	0.9300	C49—C55	1.490 (7)
C9—H9	0.9300	C50—C51	1.377 (8)
C10—H10	0.9300	C51—C52	1.385 (8)
C12—H12	0.9300	C52—C53	1.373 (9)
C13—H13	0.9300	C53—C54	1.371 (8)
C14—H14	0.9300	C55—C56	1.424 (7)
C15—H15	0.9300	C56—C57	1.439 (8)
C16—H16	0.9300	C56—C58	1.381 (7)
C17—C18	1.387 (3)	C59—C60	1.383 (8)
C17—C22	1.392 (5)	C59—C64	1.348 (8)
C17—C23	1.463 (5)	C60—C61	1.375 (8)
C18—C19	1.376 (6)	C61—C62	1.351 (9)
C19—C20	1.360 (7)	C62—C63	1.375 (9)
C20—C21	1.375 (7)	C63—C64	1.379 (9)
C21—C22	1.388 (7)	C50—H50	0.9300
C23—C24	1.437 (7)	C51—H51	0.9300
C24—C25	1.437 (7)	C52—H52	0.9300
C24—C26	1.379 (7)	C53—H53	0.9300
C27—C28	1.362 (8)	C54—H54	0.9300
C27—C32	1.372 (7)	C57—H57	0.9300
C28—C29	1.379 (10)	C58—H58	0.9300
C29—C30	1.362 (8)	C60—H60	0.9300
C30—C31	1.367 (8)	C61—H61	0.9300
C31—C32	1.393 (8)	C62—H62	0.9300
C18—H18	0.9300	C63—H63	0.9300
C19—H19	0.9300	C64—H64	0.9300
			
N2—N1—C7	104.8 (4)	C28—C29—H29	120.00
N1—N2—C10	111.8 (4)	C30—C29—H29	120.00
N1—N2—C11	118.8 (4)	C29—C30—H30	120.00
C10—N2—C11	129.4 (4)	C31—C30—H30	120.00
N4—N3—C23	105.0 (2)	C32—C31—H31	119.00
N3—N4—C26	112.5 (3)	C30—C31—H31	120.00
N3—N4—C27	119.0 (2)	C27—C32—H32	121.00
C26—N4—C27	128.5 (3)	C31—C32—H32	121.00
N6—N5—C39	105.2 (4)	C34—C33—C38	118.7 (5)
N5—N6—C43	118.9 (4)	C34—C33—C39	121.0 (5)
N5—N6—C42	112.5 (4)	C38—C33—C39	120.3 (5)
C42—N6—C43	128.6 (5)	C33—C34—C35	120.3 (6)
N8—N7—C55	104.4 (4)	C34—C35—C36	119.5 (6)
N7—N8—C58	112.6 (4)	C35—C36—C37	121.2 (6)
C58—N8—C59	128.2 (5)	C36—C37—C38	119.3 (6)
N7—N8—C59	119.0 (4)	C33—C38—C37	120.9 (6)
C2—C1—C7	120.1 (5)	N5—C39—C33	118.9 (4)
C2—C1—C6	120.1 (5)	N5—C39—C40	110.8 (4)
C6—C1—C7	119.8 (5)	C33—C39—C40	130.3 (5)
C1—C2—C3	119.5 (6)	C39—C40—C41	130.2 (5)
C2—C3—C4	121.5 (6)	C39—C40—C42	104.4 (5)
C3—C4—C5	118.8 (6)	C41—C40—C42	125.3 (5)
C4—C5—C6	120.1 (6)	O3—C41—C40	124.5 (5)
C1—C6—C5	120.1 (6)	N6—C42—C40	107.1 (5)
N1—C7—C8	110.9 (4)	N6—C43—C44	119.8 (5)
N1—C7—C1	119.7 (4)	C44—C43—C48	121.3 (5)
C1—C7—C8	129.4 (5)	N6—C43—C48	118.9 (5)
C9—C8—C10	126.7 (5)	C43—C44—C45	118.8 (6)
C7—C8—C10	104.7 (4)	C44—C45—C46	120.1 (6)
C7—C8—C9	128.6 (5)	C45—C46—C47	120.7 (6)
O1—C9—C8	125.8 (5)	C46—C47—C48	119.8 (6)
N2—C10—C8	107.9 (4)	C43—C48—C47	119.4 (5)
N2—C11—C12	119.7 (5)	C33—C34—H34	120.00
C12—C11—C16	120.7 (4)	C35—C34—H34	120.00
N2—C11—C16	119.7 (4)	C34—C35—H35	120.00
C11—C12—C13	118.4 (5)	C36—C35—H35	120.00
C12—C13—C14	121.5 (6)	C35—C36—H36	119.00
C13—C14—C15	119.4 (6)	C37—C36—H36	119.00
C14—C15—C16	120.5 (5)	C38—C37—H37	120.00
C11—C16—C15	119.6 (3)	C36—C37—H37	120.00
C1—C2—H2	120.00	C33—C38—H38	120.00
C3—C2—H2	120.00	C37—C38—H38	120.00
C2—C3—H3	119.00	C40—C41—H41	118.00
C4—C3—H3	119.00	O3—C41—H41	118.00
C5—C4—H4	121.00	C40—C42—H42	127.00
C3—C4—H4	121.00	N6—C42—H42	126.00
C4—C5—H5	120.00	C45—C44—H44	121.00
C6—C5—H5	120.00	C43—C44—H44	121.00
C5—C6—H6	120.00	C44—C45—H45	120.00
C1—C6—H6	120.00	C46—C45—H45	120.00
O1—C9—H9	117.00	C45—C46—H46	120.00
C8—C9—H9	117.00	C47—C46—H46	120.00
N2—C10—H10	126.00	C48—C47—H47	120.00
C8—C10—H10	126.00	C46—C47—H47	120.00
C11—C12—H12	121.00	C43—C48—H48	120.00
C13—C12—H12	121.00	C47—C48—H48	120.00
C12—C13—H13	119.00	C50—C49—C54	119.8 (5)
C14—C13—H13	119.00	C50—C49—C55	121.5 (4)
C13—C14—H14	120.00	C54—C49—C55	118.8 (4)
C15—C14—H14	120.00	C49—C50—C51	119.8 (5)
C16—C15—H15	120.00	C50—C51—C52	120.9 (5)
C14—C15—H15	120.00	C51—C52—C53	118.6 (6)
C15—C16—H16	120.00	C52—C53—C54	121.5 (6)
C11—C16—H16	120.00	C49—C54—C53	119.5 (5)
C18—C17—C22	117.6 (2)	N7—C55—C49	118.2 (4)
C18—C17—C23	119.7 (2)	N7—C55—C56	111.3 (4)
C22—C17—C23	122.7 (3)	C49—C55—C56	130.5 (5)
C17—C18—C19	121.6 (3)	C55—C56—C57	128.9 (5)
C18—C19—C20	120.4 (5)	C55—C56—C58	104.7 (4)
C19—C20—C21	119.5 (5)	C57—C56—C58	125.7 (5)
C20—C21—C22	120.8 (5)	O4—C57—C56	125.1 (5)
C17—C22—C21	120.2 (4)	N8—C58—C56	107.0 (4)
C17—C23—C24	129.6 (4)	N8—C59—C60	120.2 (5)
N3—C23—C24	111.1 (4)	N8—C59—C64	119.3 (5)
N3—C23—C17	119.3 (4)	C60—C59—C64	120.5 (5)
C25—C24—C26	126.2 (5)	C59—C60—C61	118.4 (5)
C23—C24—C26	104.1 (4)	C60—C61—C62	122.1 (6)
C23—C24—C25	129.0 (5)	C61—C62—C63	118.4 (6)
O2—C25—C24	124.7 (5)	C62—C63—C64	120.8 (6)
N4—C26—C24	107.3 (4)	C59—C64—C63	119.8 (6)
N4—C27—C28	118.9 (4)	C49—C50—H50	120.00
C28—C27—C32	120.9 (5)	C51—C50—H50	120.00
N4—C27—C32	120.2 (4)	C50—C51—H51	120.00
C27—C28—C29	119.8 (5)	C52—C51—H51	120.00
C28—C29—C30	120.5 (6)	C51—C52—H52	121.00
C29—C30—C31	119.5 (6)	C53—C52—H52	121.00
C30—C31—C32	120.9 (5)	C52—C53—H53	119.00
C27—C32—C31	118.4 (5)	C54—C53—H53	119.00
C17—C18—H18	119.00	C49—C54—H54	120.00
C19—C18—H18	119.00	C53—C54—H54	120.00
C20—C19—H19	120.00	O4—C57—H57	118.00
C18—C19—H19	120.00	C56—C57—H57	117.00
C19—C20—H20	120.00	N8—C58—H58	127.00
C21—C20—H20	120.00	C56—C58—H58	127.00
C22—C21—H21	120.00	C59—C60—H60	121.00
C20—C21—H21	120.00	C61—C60—H60	121.00
C17—C22—H22	120.00	C60—C61—H61	119.00
C21—C22—H22	120.00	C62—C61—H61	119.00
O2—C25—H25	118.00	C61—C62—H62	121.00
C24—C25—H25	118.00	C63—C62—H62	121.00
N4—C26—H26	126.00	C62—C63—H63	120.00
C24—C26—H26	126.00	C64—C63—H63	120.00
C29—C28—H28	120.00	C59—C64—H64	120.00
C27—C28—H28	120.00	C63—C64—H64	120.00
			
C7—N1—N2—C10	0.2 (5)	C17—C18—C19—C20	−0.2 (7)
C7—N1—N2—C11	179.9 (4)	C18—C19—C20—C21	−0.3 (8)
N2—N1—C7—C1	−179.3 (4)	C19—C20—C21—C22	−0.2 (9)
N2—N1—C7—C8	−1.0 (6)	C20—C21—C22—C17	1.1 (8)
N1—N2—C10—C8	0.7 (6)	N3—C23—C24—C25	171.9 (5)
C11—N2—C10—C8	−178.9 (5)	C17—C23—C24—C25	−9.1 (9)
N1—N2—C11—C12	−179.0 (5)	C17—C23—C24—C26	−179.5 (5)
N1—N2—C11—C16	−0.4 (6)	N3—C23—C24—C26	1.5 (6)
C10—N2—C11—C12	0.6 (8)	C26—C24—C25—O2	−12.8 (8)
C10—N2—C11—C16	179.3 (4)	C23—C24—C26—N4	−1.4 (5)
N4—N3—C23—C24	−0.9 (4)	C23—C24—C25—O2	178.7 (5)
N4—N3—C23—C17	180.0 (3)	C25—C24—C26—N4	−172.2 (5)
C23—N3—N4—C26	0.0 (4)	C28—C27—C32—C31	2.2 (8)
C23—N3—N4—C27	−176.8 (3)	N4—C27—C28—C29	179.1 (5)
C27—N4—C26—C24	177.4 (4)	N4—C27—C32—C31	−178.6 (4)
C26—N4—C27—C28	−162.0 (5)	C32—C27—C28—C29	−1.7 (9)
N3—N4—C27—C28	14.2 (6)	C27—C28—C29—C30	1.2 (10)
N3—N4—C27—C32	−165.0 (4)	C28—C29—C30—C31	−1.2 (10)
N3—N4—C26—C24	1.0 (5)	C29—C30—C31—C32	1.8 (9)
C26—N4—C27—C32	18.8 (7)	C30—C31—C32—C27	−2.3 (8)
C39—N5—N6—C42	−0.6 (6)	C38—C33—C34—C35	1.7 (8)
N6—N5—C39—C40	0.5 (6)	C34—C33—C38—C37	0.2 (8)
N6—N5—C39—C33	−179.3 (4)	C39—C33—C38—C37	−179.0 (5)
C39—N5—N6—C43	176.9 (4)	C39—C33—C34—C35	−179.2 (5)
N5—N6—C43—C48	0.6 (7)	C38—C33—C39—C40	−152.4 (5)
N5—N6—C43—C44	−179.1 (5)	C38—C33—C39—N5	27.4 (7)
C42—N6—C43—C48	177.7 (5)	C34—C33—C39—N5	−151.8 (5)
C42—N6—C43—C44	−1.9 (8)	C34—C33—C39—C40	28.5 (8)
C43—N6—C42—C40	−176.8 (5)	C33—C34—C35—C36	−1.4 (9)
N5—N6—C42—C40	0.5 (6)	C34—C35—C36—C37	−0.7 (10)
C55—N7—N8—C58	0.7 (6)	C35—C36—C37—C38	2.6 (10)
C55—N7—N8—C59	−175.6 (5)	C36—C37—C38—C33	−2.3 (9)
N8—N7—C55—C56	−1.1 (6)	N5—C39—C40—C41	−175.4 (5)
N8—N7—C55—C49	−179.3 (4)	C33—C39—C40—C42	179.5 (5)
C58—N8—C59—C60	21.8 (8)	N5—C39—C40—C42	−0.2 (6)
N7—N8—C59—C60	−162.6 (5)	C33—C39—C40—C41	4.3 (9)
N7—N8—C59—C64	19.0 (8)	C39—C40—C41—O3	−174.8 (6)
N7—N8—C58—C56	0.1 (6)	C41—C40—C42—N6	175.3 (5)
C58—N8—C59—C64	−156.6 (6)	C42—C40—C41—O3	10.9 (9)
C59—N8—C58—C56	175.9 (5)	C39—C40—C42—N6	−0.2 (6)
C6—C1—C7—C8	43.6 (8)	C44—C43—C48—C47	−0.1 (9)
C7—C1—C2—C3	179.0 (5)	N6—C43—C48—C47	−179.7 (5)
C2—C1—C7—C8	−136.3 (6)	N6—C43—C44—C45	179.2 (5)
C6—C1—C7—N1	−138.5 (6)	C48—C43—C44—C45	−0.4 (9)
C6—C1—C2—C3	−0.9 (8)	C43—C44—C45—C46	0.7 (9)
C2—C1—C6—C5	0.1 (9)	C44—C45—C46—C47	−0.5 (10)
C2—C1—C7—N1	41.7 (7)	C45—C46—C47—C48	0.0 (10)
C7—C1—C6—C5	−179.8 (5)	C46—C47—C48—C43	0.3 (9)
C1—C2—C3—C4	0.7 (8)	C54—C49—C50—C51	1.7 (8)
C2—C3—C4—C5	0.4 (9)	C55—C49—C50—C51	−177.9 (5)
C3—C4—C5—C6	−1.2 (10)	C50—C49—C54—C53	−1.5 (9)
C4—C5—C6—C1	1.0 (10)	C55—C49—C54—C53	178.1 (6)
C1—C7—C8—C10	179.5 (5)	C50—C49—C55—N7	143.5 (5)
N1—C7—C8—C9	−176.1 (5)	C50—C49—C55—C56	−34.3 (8)
C1—C7—C8—C9	2.1 (9)	C54—C49—C55—N7	−36.1 (7)
N1—C7—C8—C10	1.4 (6)	C54—C49—C55—C56	146.2 (6)
C10—C8—C9—O1	4.9 (10)	C49—C50—C51—C52	−1.1 (9)
C9—C8—C10—N2	176.3 (5)	C50—C51—C52—C53	0.2 (10)
C7—C8—C9—O1	−178.2 (6)	C51—C52—C53—C54	0.1 (10)
C7—C8—C10—N2	−1.2 (6)	C52—C53—C54—C49	0.6 (10)
N2—C11—C12—C13	179.3 (5)	N7—C55—C56—C57	172.2 (5)
C12—C11—C16—C15	−0.7 (6)	N7—C55—C56—C58	1.2 (6)
N2—C11—C16—C15	−179.3 (4)	C49—C55—C56—C57	−10.0 (9)
C16—C11—C12—C13	0.7 (8)	C49—C55—C56—C58	179.1 (5)
C11—C12—C13—C14	0.4 (9)	C55—C56—C57—O4	−177.9 (6)
C12—C13—C14—C15	−1.5 (10)	C58—C56—C57—O4	−8.7 (9)
C13—C14—C15—C16	1.5 (8)	C55—C56—C58—N8	−0.7 (6)
C14—C15—C16—C11	−0.4 (7)	C57—C56—C58—N8	−172.1 (5)
C23—C17—C18—C19	178.6 (4)	N8—C59—C60—C61	−177.6 (5)
C18—C17—C22—C21	−1.5 (6)	C64—C59—C60—C61	0.8 (9)
C22—C17—C18—C19	1.1 (5)	N8—C59—C64—C63	178.1 (6)
C22—C17—C23—N3	145.3 (4)	C60—C59—C64—C63	−0.3 (9)
C22—C17—C23—C24	−33.7 (7)	C59—C60—C61—C62	−1.2 (9)
C18—C17—C23—C24	149.0 (4)	C60—C61—C62—C63	1.1 (10)
C23—C17—C22—C21	−178.9 (5)	C61—C62—C63—C64	−0.6 (10)
C18—C17—C23—N3	−32.1 (5)	C62—C63—C64—C59	0.2 (10)

### Hydrogen-bond geometry (Å, °)

**Table:**

Cg5 is the centroid of the C17–C22 ring.

**Table:**

*D*—H···*A*	*D*—H	H···*A*	*D*···*A*	*D*—H···*A*
C10—H10···O2^i^	0.93	2.38	3.299 (5)	168
C12—H12···O2^i^	0.93	2.45	3.373 (6)	175
C26—H26···O1^i^	0.93	2.38	3.298 (6)	169
C32—H32···O1^i^	0.93	2.55	3.423 (7)	156
C42—H42···O3^ii^	0.93	2.36	3.287 (7)	173
C44—H44···O3^ii^	0.93	2.57	3.495 (8)	175
C58—H58···O4^iii^	0.93	2.44	3.353 (6)	165
C60—H60···O4^iii^	0.93	2.49	3.330 (8)	150
C2—H2···Cg5^iv^	0.93	2.86	3.689 (7)	149

Symmetry codes: (i) −*x*+1, −*y*+1, −*z*+1; (ii) −*x*+1, −*y*+1, −*z*; (iii) −*x*, −*y*+1, −*z*; (iv) *x*+1, *y*, *z*.
